# Modern Sociology

**Published:** 1902-05-31

**Authors:** 


### Modern Sociology.

### SPECIAL SCHOOLS UNDER THE LONDON SCHOOL BOARD.

### V.—For Cripples.

The Schools for Cripples are among the latest developments of the London School Board, and owe their introduction to the novelist, Mrs. Humphry Ward, through whose efforts a school for crippled and invalid children was opened in February, 1899, at the Passmore Edwards Settlement in Tavistock Place.

Its history is simple. There had been for some time scattered classes for invalid children in London, maintained by private effort; Miss Pilcher had a class in Stepney, Miss Sparks in South-east London. The former grew out of the Invalid Children's Aid Association, the latter was carried on in connection with the Southwark Women's Settlement.

The Settlement in Tavistock Place was the possessor of a beautiful building used chiefly in the evening; Mrs. Humphry Ward and others thought it would be a good thing to make some use of the rooms by day also. They therefore decided to open a class for invalid children, whose sad lot and hopeless future appealed forcibly to the sympathies of the novelist. "It soon became plain," she says, "that if such a class were begun, it ought (*a*) to be placed under the London School Board; (*b*) to be provided with more complete appliances for the transport and medical care of the children than had yet been attempted." A small sub-committee was formed, composed of the district visitors for the Invalid Children's Aid Association, the head of the Metropolitan Nursing Association, and a doctor specially versed in the diseases of children. A list was obtained of children excused from attendance at school on the score of health, names of suitable cases were supplied by the neighbouring hospitals, and from these, and the information given by the Invalid Visitors, after much careful sifting, a schedule of 25 cases was made out and sent to the Board. "They met us at once," says Mrs. Ward; "the scheme passed quickly through, and by February a mistress had been appointed, special furniture had been provided, and the school opened as one of the Board's Special Classes."

The sub-committee had in the meantime appointed a nurse and ordered an ambulance. The latter was to have been paid for on the hire system, but Sir Thomas and Lady Barlow gave this at a cost of £100.

The school, which opened with 25 children, soon increased the number of its scholars to 45, with an average attendance of from 32 to 38. Here are some of the diseases from which the pupils suffered:—Hip disease, spinal caries, heart disease, infantile paralysis, tuberculous knee, hydrocephalus, abscesses in groin, rickets. All wanted special care, being, as a rule, in weak health, even if the disease from which they suffered was no longer acute. Many had to lie down, or at least to recline in a specially made chair.

In the following year, the London School Board made an inquiry about the need for such schools, and after receiving evidence and sifting it, resolutions were passed, under the chairmanship of Mr. Graham Wallas, recommending the provision of a certain number of centres for physically defective children, and a clause was inserted recommending "that children of normal intelligence be not taught with mentally defective children." It had been thought by some that provision might be made for these children either in classes for the mentally deficient or in infant schools; while others maintained that, with a little extra care, all who could attend school at all could attend the ordinary school. With these two opinions Mrs. Humphry Ward is in disagreement, and that the Board has, to a certain extent at any rate, adopted her views, is evidenced by the fact that there are now four centres, and that a special school is to be built in Whitechapel for a class at present accommodated in Toynbee Hall.

It is sufficiently evident that many of these children are quite up to the normal standard of intelligence and that some are above it. They may be, and often are, backward through neglect, but those who have to do with them bear testimony to the remarkable progress they make when once regular attendance at school becomes possible. "God has taken away my legs, but He hasn't taken away my head," one child said, crying bitterly, after a day spent at one of the centres for the mentally deficient. "Their intelligence," says Mrs. Ward, "is their only capital." And for this reason, also, it seems unadvisable to leave a cripple child in the infant school, where the teaching is as far below its mental capacity as the games in the playground are beyond its bodily strength.

The scheme of instruction is very much on kindergarten lines. "Occupations" play a large part, and drawing, brushwork, and needlework are important. Some of the girls at Tavistock Place have done some embroidery which gives great promise, and it seems quite reasonable to suppose that openings may be found for them in art needlework shops. They seem to enjoy their lessons, and when one realises what being a cripple must mean to some of these children, how, while the brothers and sisters are at school and the parents at work, the lame child may be shut into a room by itself for safety, and how limited its little life must be in every way, one cannot help feeling that the Board is doing a beneficent work in thus giving the cripples of London a chance. It is estimated that there are some 1,450 of these children, 850 of whom are not attending any school, and this number is below rather than above the total. Mrs. Humphry Ward puts the number at about 2,000, "of whom a large proportion might be prepared for a wage-earning life."

Many of the children are, in medical parlance, stretcher-cases, and others are unable to walk; they are fetched to school and taken home again each day by the nurse in an ambulance (both provided by the Board).

Quite as important as the lessons is the food. The children remain at school till the afternoon, and the majority pay twopence for dinner. This is an excellent wholesome meal, and the twopences nearly cover the cost at Tavistock Place; at Toynbee Hall the nurse assured us that she "made the dinners pay." Possibly the cost of provisions is lower in Whitechapel. Great physical improvement has been noticed in the children after some months of this regular wholesome diet. Dr. Gwyther writes after visiting the Tavistock Place School: "There has been a distinct improvement in the general health of most of the children." Here, too, there is a beautiful garden, which is a great delight and resource in the play-hour; and it has been suggested that a good plan would be to take some of the small old-fashioned houses with gardens which still exist in some parts of London, rather than to place the special centre in the ordinary school building, where an asphalt playground is a poor substitute for the garden, even when, as at Paddington, strips of cocoa-nut matting are laid down.

The whole of the Board's work in the direction of invalid schools is, however interesting, too new to enable an opinion to be formed as to its ultimate results; but it is noteworthy that at Tavistock Place five children have been placed out in different trades or at technical classes under the Board.

A special committee to consider the after training of the children was appointed quite recently; this, though it includes some representatives of the Board, is an independent organisation. The results of its deliberations will be watched with interest.

